# Prevalence of metabolic syndrome and its determinants among Iranian adults: evidence of IraPEN survey on a bi-ethnic population

**DOI:** 10.1038/s41598-019-44486-8

**Published:** 2019-05-28

**Authors:** Leila Jahangiry, Leila Khosravi-far, Parvin Sarbakhsh, Ahmad Kousha, Rasool EntezarMahdi, Koen Ponnet

**Affiliations:** 1 Tabriz Health Services Management Research Center, Tabriz University of Medical Sciences, Tabriz, Iran; 2 Department of Health Education and Health Promotion, Faculty of Health, Tabriz University of Medical Sciences, Tabriz, Iran; 3 Department of Epidemiology and Biostatistics, Faculty of Health, Tabriz University of Medical Sciences, Tabriz, Iran; 4 Department of Biostatistics and Epidemiology, School of Medicine, Urmia University of Medical Sciences, Urmia, Iran; 5 Faculty of Political and Social Sciences, IMEC-MICT, Ghent University, Ghent, Belgium

**Keywords:** Diseases, Health care, Risk factors

## Abstract

Metabolic syndrome (MetS) is a growing public health concern worldwide. It has been demonstrated that individuals with MetS are at an increased risk of cardiovascular events and diabetes. We aimed to investigate the prevalence of MetS and its components among Turkic and Kurds ethnic groups in a bi-ethnic (Turk and Kurd) population. This cross-sectional study is part of the national health transformation plan created in response to the emerging epidemic of non-communicable diseases (Iran’s Package of Essential Non-communicable Disease study), launched in 2014 in Naqadeh, Iran. In total, 3506 participants aged 30–70 years were randomly included in the study from urban and rural regions. Cardio-metabolic risk factors related to MetS diagnosis and other related sociodemographic factors were assessed for men and women in both the Turk and the Kurd population. Multivariate logistic regressions were applied to identify MetS -associated factors among both the Turk and the Kurd population. The mean (SD) age of the participants was 49.6 (12.3) years. Of the participants, 56.2% (n = 1969) were women, and 43.8% (n = 1537) were men. Three-fifths of the participants were Turk (60.3%, n = 1751). The overall prevalence of MetS was 37.05%, with a higher prevalence in women (49.8% versus 24.3% in men). The prevalence of MetS and its components among Turk people (41.6%) were significantly higher than that among Kurd people (33.9%) (p < 0.0001). In addition, the prevalence of MetS was higher among women, urban, and older people for both ethnicities. Strong associations were found between MetS prevalence and being older, being female, being overweight, being obese, having a higher waist-to-hip ratio, and having a history of diabetes and cardiovascular disease (CVD) in the family for both Turks and Kurds. The raised waist circumference (WC) is the most prevalent MetS component for Turk men and women. Meanwhile, the most prevalent MetS component for Kurd participants is low high-density lipoprotein for women and a raised WC for men. Significant differences were found between Kurdish men and women for all components, except for a raised WC and a raised fasting blood glucose (p < 0.05). Because the Iranian population features multiple ethnicities, the recognition of the prevalence of MetS components is a major step in establishing intervention strategies for modifying cardio-metabolic risk factors based on the population ethnicities and their socio-demographic, cultural, and lifestyle factors. We recommend future studies for planning an efficient and sustainable health education and promotion program to halt MetS prevalence.

## Introduction

Metabolic syndrome (MeTS) is a growing public health concern worldwide. It is a cluster of related MeTS risk factors that are characterized by abdominal obesity, increased triglycerides (TG), hypertension, an elevated fasting blood glucose (FBG) level, and reduced high-density lipoprotein (HDL) cholesterol^1^. Various definitions for the diagnosis of MetS currently exist^2,3^. The National Cholesterol Education Program Adult Treatment Panel (ATP) III defines METS as having three or more of the following conditions: an HDL of <40 mg/dl in men and <50 mg/dl in women, a systolic/diastolic blood pressure (BP) of ≥130/85 mm Hg, a triglyceride level at least 150 mg/dl, an FBG level of ≥100 mg/dl, and a waist circumference (WC) > 102 cm in men and >88 cm in women^4^.

The International Diabetes Federation (IDF) proposed another definition, viewing central obesity as an essential part of METS^3^. Meanwhile, the American Heart Association/National Heart Lung and Blood Institute focuses on the accumulation of risk factors and does not place emphasis on central obesity^5^. An important step for MetS control is designing tailored interventions to various ethnic groups and to assess MetS elements with people from different ethnic backgrounds. In several countries, ethnic differences in the prevalence of cardiovascular diseases risk factors have been described^6–8^. However, only limited attention has been paid to ethnic differences in the prevalence of MetS and its components. A systematic review of Dalvand *et al*.^9^ has demonstrated an emerging high prevalence of MetS in general and in particular among Iranian people.

According to the IDF, central obesity is defined based on ethnicity-specific criteria, and for various ethnic groups, a cut-off for central obesity should be applied based on the population features^10^. Several studies in Iran have recommended that an optimal WC for the Iranian population is greater than 90 cm^11,12^. It has been demonstrated that individuals with METS are at an increased risk of cardiovascular events and diabetes^13^. A meta-analysis of 34 studies with 26,609 participants showed that 4.8–7% of young adults had METS between 2004 to 2015^14^. The prevalence of METS among Iranians is increasing. The Tehran Lipid and Glucose Study (TLGS) reported MetS for women at 42% and for men at 24%^15^.

Iran is a multi-ethnic country with various ethnic groups, including Persians, Kurds, Lurs, Arabs, Turkics, Baluchi, and Turkmen. Turkics or Azeris are the second-largest ethnic group in Iran, making up an estimated 16% of the total population, and can be found in the northwestern part of the country. Meanwhile, Kurds are the third-largest ethnic groups in Iran and make up 10% of the population^16^. Naqadeh is a city in the western part of Azebaijan province in Iran and features the two largest ethnic groups of Trukic and Kurds^17^.

In this study, we aimed to investigate the prevalence of MetS and its components among the Turkic and Kurd ethnic groups in Naqadeh. MetS as a cluster of several risk factors is a useful metric for measuring the CDV health of the population in Naqadeh. Additionally, estimates of MetS prevalence will prove useful in evaluating the effectiveness of population-based lifestyle interventions across Iran’s Package of Essential Non-communicable Disease study (IraPEN program).

## Methods

### About iraPEN program in naqadeh

IraPEN is part of the national health transformation plan created in response to the emerging epidemic of non-communicable diseases, launched in 2014 by the Iranian Ministry of Health and Medical Education, to provide universal health coverage, including access to noncommunicable disease prevention and care as well as mental health services^18^. Naqadeh is a city in the western part of Azebaijan province, Iran, and features the two largest ethnic groups of Turks and Kurds and a population of 127,671. IraPEN has been successfully piloted in Iran’s four main districts, and its nationwide scale-up has begun in at least one district per province. All the methods were performed in accordance with the relevant guidelines and regulations.

### Participants

Participants were all people 30–70 years old from urban and rural regions. Participants were recruited using household health files from health centers in urban areas and health houses in rural regions.

### MeTS measurements

Prevalence of MetS was defined based on the National Cholesterol Education Program (NCEP) Adult Treatment Panel III (ATP III) as the presence of three or more of the following criteria (except for waist circumference which was defined as ≥90 cm for both genders for Iranian population): triglycerides level <150 mg dl^−1^, HDL level <40 mg dl^−1^ in men and <50 mg dl^−1^ in women, systolic/diastolic blood pressure 130/85 mmHg or higher and fasting blood glucose level 110 mg dl^−1^ or higher^4^. The International Diabetes Federation (IDF) has declared that WC is both a gender and ethnic-group specific indicator and that using this approach may provide a better assessment of obesity-related risk globally than using a single cut-off point^19^. The current definitions of central adiposity or waist circumference are based on data from western populations. However, several studies in Iran have suggested that a more realistic waist circumference for Iranians is >90 cm for both genders^11,12,20^. Blood sampling was collected for measurements of total cholesterol, triglycerides, LDL cholesterol, HDL-cholesterol, and fasting blood glucose for all participants. Overnight fasting for 12–14 hours was needed before blood sampling. Venous blood samples (5 ml) were collected. Fasting blood glucose (FBG) was measured by using the glucose oxidase method (intra- and inter assay coefficients of variation 2.1% and 2.6%, respectively). Body Mass Index (BMI) was measured by individual’s weight divided by the square of the height. Waist-to-hip ratio was calculated by waist circumference divided by hip circumference.

The WC was evaluated using a measuring tape to the nearest 0.1 cm. The weight of an individual dressed in light clothing without shoes was recorded each time using a calibrated scale to the nearest 0.1 kg. Height was measured without shoes using a stadiometer to the nearest 0.1 cm. BP was measured with a mercury sphygmomanometer twice in the same arm after the individual had been seated at rest for 10–15 minutes. The systolic and diastolic BP measurements were the mean of the two readings. Blood samples were collected for the measurement of total cholesterol, TG, low-density lipoprotein cholesterol, HDL cholesterol, and FBG for all participants. FBG was measured using the glucose oxidase method (intra- and inter-assay coefficients of variation of 2.1% and 2.6%, respectively).

All participants also responded to sociodemographic questions about their gender, age, marital status (single, married, divorced/widowed), educational qualifications (illiterate, primary, secondary, university degree), personal history, and family history of diabetes and hypertension (yes/no).

### Statistical analysis

Statistical analyses were performed with Statistical Package for Social Science (SPSS 18 for windows, SPSS Inc.® headquarter, Chicago, USA). Normality of data was analyzed by Kolmogorov-Smirnov test. Continuous and discrete variables are presented with mean and ± SD, and number and percentage, respectively. Chi-square analyses were used to test the difference between biochemical variables between two groups. Multiple logistic regressions analysis was used to examine the associations between risk factors of metabolic syndrome and socio demographic factors as independent and dependent variables, respectively. Adjusted odds ratio and 95% confidence intervals were calculated for all metabolic syndrome parameters. P values less than 0.05 were regarded as statistically significant.

### Ethical considerations

The ethics committee of Tabriz University of Medical Sciences approved the study (IRTBZMED.REC.1396.965). A signed informed consent form explaining the study purposes was obtained from each participant.

## Results

Of 3691 participants, 3506 were evaluated, and 185 records were removed because of incomplete demographic or clinical information to assess accurate MetS measurements.

### Demographic characteristics

The mean (SD) age of the participants was 49.6 (12.3) years. A total of 56.2% of participants were women, and 43.8 were men. About 35% of the participants were illiterate, and about two-thirds lived in urban areas. Regarding the participants’ ethnicity, three-fifths of them were Turks (60.3 Turks vs. 39.7 Kurds). See Table 1 for an overview.

**Table 1:** Prevalence of metabolic syndrome according to demographic characteristics of population.

	Overall	Metabolic syndrome		
Sex				<0.0001
Male	1537 (43.8)	374 (24.3)	1163 (75.7)	
Female	1969 (56.2)	980 (49.8)	989 (50.2)	
Age				<0.0001
30–39	712 (20.3)	188 (26.4)	524 (73.6)	
40–49	1161 (33.1)	408 (35.1)	753 (64.9)	
50–59	916 (26.1)	417 (54.5)	499 (54.5)	
60–69	465 (13.3)	232 (49.9)	233 (50.1)	
70+	252 (7.2)	109 (8.1)	143 (6.6)	
Education				<0.0001
Illiterate	1223 (35.0)	584 (47.8)	639 (52.2)	
Elementary	947 (27.1)	367 (38.8)	580 (61.2)	
Guidance school	530 (15.2)	165 (31.1)	365 (68.9)	
High school	578 (16.5)	172 (29.8)	406 (70.2)	
University	217 (6.2)	63 (29.0)	154 (71.0)	
Ethnicity				<0.0001
Turk	1751 (60.3)	729 (41.6)	57.3 (58.4)	
Kurd	1152 (39.7)	391 (33.9)	761 (66.1)	
Marital status				<0.0001
Married	3152 (89.9)	1183 (37.4)	1969 (62.5)	
Single	91 (2.6)	33 (36.3)	58 (63.7)	
Widowed	247 (7.0)	134 (54.3)	113 (45.7)	
Divorced	13 (0.4)	3 (23.1)	10 (76.9)	
Locality				<0.0001
Urban	2394 (68.3)	991 (41.4)	1403 (58.6)	
Rural	1112 (31.7)	363 (32.6)	749 (67.4)	
Smoking (yes)	309 (8.8)	70 (22.7)	239 (77.3)	<0.0001
Alcohol use (yes)	15 (0.4)	4 (26.7)	11 (73.3)	<0.0001
Family history of diabetes (yes)	658 (18.8)	302 (45.9)	356 (54.1)	<0.0001
Having hypertension (yes)	568 (16.2)	367 (64.6)	201 (35.4)	<0.0001
Having diabetes	261 (7.4)	207 (79.3)	54 (20.7)	<0.0001

### Prevalence of metabolic syndrome

The overall prevalence of MetS was 37.05%, with a higher prevalence in women (49.8% versus 24.3% in men). There was a progressive increase in the prevalence of MetS with the increasing age of the participants (particularly those 50–59 years old). The percentages of the prevalence of MetS among Turks and Kurds was 41.6% and 33.9%, respectively. About half of the illiterate participants (47.8%) had MetS, and this percentage decreased with the increase of the education level. Also, about two-fifths of the participants in urban areas have MetS.

### Prevalence of metabolic syndrome components

Table 2 depicts the prevalence of MetS components according to the ethnicities and sociodemographic characteristics of the study population. By comparing MetS components between both Turk and Kurd participants, we found that a raised WC is the most prevalent MetS component for Turk men and women and that the most prevalent MetS component for Kurd participants is low HDL for women and a raised WC for men. Significant differences were found between Kurdish men and women for all components except for a raised WC and raised FBS (p < 0.05).

**Table 2:** Prevalence of metabolic syndrome components (ATP III) according to social characteristics of participants of Turk and Kurd ethnicities.

	Turk; N (%)					Kurd; N (%)				
	Raised BP	Raised WC	Raised FBS	Raised TG	Low HDL-C	Raised BP	Raised WC	Raised FBS	Raised TG	Low HDL-C
**Sex**										
Male	236 (31.2)	548 (72.8)	182 (24.2)	357 (48.1)	390 (52.1)	151 (26.0)	414 (71.5)	105 (18.6)	261 (46.4)	321 (57.2)
Female	410 (38.6)	825 (77.5)	336 (31.8)	440 (41.9)	805 (76.6)	205 (32.2)	464 (73.0)	130 (20.9)	245 (39.7)	490 (79.4)
P-value	<0.0001	0.022	<0.0001	0.010	<0.0001	<0.018	0.572	0.311	0.021	<0.0001
**Age**										
30–39	37 (13.7)	186 (67.9)	18 (7.1)	101 (40.6)	193 (76.9)	44 (11.4)	265 (68.1)	16 (4.5)	121 (34.6)	259 (73.8)
40–49	135 (22.7)	440 (73.6)	135 (22.5)	277 (46.6)	417 (70.0)	79 (21.9)	261 (72.5)	74 (20.4)	166 (46.0)	251 (69.5)
50–59	226 (42.9)	428 (80.9)	195 (36.6)	235 (44.5)	332 (62.8)	104 (42.1)	184 (75.1)	80 (32.3)	115 (46.2)	158 (64.2)
60–69	147 (54.9)	215 (81.1)	117 (43.5)	121 (45.3)	151 (56.1)	75 (52.4)	117 (83.0)	49 (35.0)	69 (49.3)	90 (64.3)
70+	101 (64.7)	104 (68.4)	53 (34.2)	63 (41.2)	102 (66.2)	54 (67.5)	51 (63.8)	16 (20.0)	35 (43.8)	53 (66.3)
P-value	<0.0001	<0.0001	<0.0001	0.491	<0.0001	<0.0001	0.004	<0.0001	0.005	0.87
**Education**										
Illiterate	295 (50.9)	463 (80.2)	229 (39.1)	248 (42.9)	385 (66.2)	198 (41.3)	362 (75.6)	127 (26.7)	215 (45.5)	332 (70.2)
Elementary	167 (33.3)	370 (74.1)	131 (26.3)	221 (44.8)	334 (67.6)	62 (20.2)	212 (68.6)	45 (15.2)	134 (45.3)	213 (72.4)
Guidance school	80 (28.3)	211 (74.3)	66 (23.5)	121(64.0)	183 (65.6)	39 (22.5)	124 (72.1)	29 (17.2)	68 (40.7)	115 (68.9)
High school	74 (23.5)	234 (73.4)	69 (22.3)	144 (46.8)	208 (67.3)	40 (20.7)	137 (71.7)	25 (13.5)	63 (34.4)	112 (61.2)
University	29 (21.5)	93 (68.4)	23 (17.3)	61 (46.2)	82 (62.1)	16 (27.1)	39 (67.2)	9 (16.4)	23 (41.8)	36 (65.5)
P-value	<0.0001	0.016	<0.0001	0.830	0.805	<0.0001	0.245	<0.0001	0.105	0.113
**Marital status**										
Married	522 (32.9)	1206 (75.8)	427 (27.0)	709 (45.3)	1042 (66.3)	308 (27.5)	812 (72.7)	208 (19.1)	465 (42.9)	743 (68.5)
Single	13 (27.1)	27 (56.3)	11 (23.9)	20 (43.5)	35 (76.1)	11 (25.6)	27 (62.8)	11 (26.8)	17 (41.5)	29 (74.4)
Widowed	108 (63.2)	132 (78.6)	77 (45.3)	65 (38.5)	108 (63.5)	35 (67.3)	38 (73.1)	16 (30.8)	24 (46.2)	38 (73.1)
Divorced	3 (27.3)	8 (72.7)	3 (27.3)	3 (27.3)	10 (90.9)	1 (50.0)	1 (50)	0 (0.0)	0 (0.0)	1 (50.0)
P-value	<0.0001	0.014	<0.0001	0.236	0.137	<0.0001	0.468	0.109	0.623	0.714
**Locality**										
Rural	169 (30.6)	380 (69.2)	125 (22.8)	230 (42.6)	350 (64.5)	80 (19.4)	281 (68.2)	59 (14.5)	171 (42.2)	289 (71.5)
Urban	477 (37.7)	993 (78.3)	393 (31.1)	567 (45.3)	845 (67.3)	276 (34.3)	597 (74.3)	176 (22.6)	335 (43.2)	522 (67.4)
P-value	0.004	45.3	45.3	0.286	0.245	<0.0001	0.024	0.001	0.741	0.150
Smoking (yes)	39 (22.9)	120 (70.6)	43 (25.1)	86 (63.2)	105 (61.4)	19 (20.0)	58 (61.1)	17 (17.9)	50 (52.6)	60 (63.8)
Alcohol use (yes)	4 (80.0)	6 (85.7)	0 (0.0)	2 (28.6)	4 (57.1)	1 (16.7)	3 (50.0)	1 (16.7)	5 (83.3)	4 (66.7)
**BMI (kg/m** ^**2**^ **)**										
Normal or under weight	77 (24.9)	98 (31.9)	59 (91.8)	102 (35.1)	171 (58.0)	54 (18.4)	98 (33.3)	37 (13.3)	91 (33.0)	181 (65.6)
Overweight	249 (33.2)	557 (74.1)	208 (27.7)	333 (44.6)	490 (65.5)	139 (27.4)	396 (78.1)	97 (19.8)	221 (45)	330 (67.3)
Obese	320 (42.2)	718 (94.6)	251 (32.9)	362 (48.0)	534 (70.6)	163 (39.1)	384 (92.8)	101 (24.3)	194 (47.0)	300 (72.8)
P-value	<0.0001	<0.0001	<<0.0001	0.001	<0.0001	<0.0001	<0.0001	0.002	0.001	0.086

*Note*. BP: blood pressure; WC: waist circumference; FBS: fasting blood sugar; TG: triglycerides; HDL-C: high-density lipoprotein cholesterol.

The prevalence of a raised BP increases significantly by age among both the Turk and the Kurd people, and people 60–69 years of age have the most prevalent WC. By comparing the components based on education level, the WC is the most prevalent among illiterate Turk and Kurd participants. Also, an increased WC is the most prevalent component among rural and urban Turk people, and low HDL is the most prevalent among both rural and urban Kurds.

Based on the categorizing of the BMI into three groups (normal or underweight, overweight, and obese), the BMI is the most prevalent MetS component for obese Turks and Kurds. The association of metabolic syndrome and sociodemographic characteristics was assessed with multi-variate analyses (see Table 3) for both Turks and Kurds separately. Among both ethnicities, being a female Turk [OR 3.44, 95% CI: 2.58–4.58; p < 0.0001], a female Kurd [OR 2.8, 95% CI: 1.92–4.08; p < 0.0001], increasing in age, having overweight or obesity, having a higher waist-to-hip ratio, and having a history of diabetes and CVD in the family showed strong associations with MetS.

**Table 3:** Socio-demographic predictors of metabolic syndrome.

	Turk		Kurd	
	OR (CI 95%)	P-value	OR (CI 95%)	P-value
**Sex**				
Male	Ref	Ref	Ref	Ref
Female	3.44 (2.58–4.58)	<0.0001	2.8 (1.92–4.08)	<0.0001
**Age**				
30–39	Ref	Ref	Ref	Ref
40–49	1.59 (1.06–2.38)	0.025	2.33 (1.36–3.99)	0.02
50–59	2.7 (1.79–4.2)	<0.0001	3.90 (2.15–7.06)	<0.0001
60–69	3.89 (2.3–6.5)	<0.0001	4.4 (2.25–7.55)	<0.0001
70+	5.1 (2.77–9.44)	<0.0001	3.32 (1.46–7.55)	<0.004
**Education**				
Illiterate	Ref	Ref	Ref	Ref
Elementary	1.27 (0.90–1.80)	0.169	0.90 (0.57–1.41)	0.653
Guidance school	0.986 (0.60–1.35)	0.944	0.71 (0.38–1.33)	0.291
High school	0. 904 (0.60–1.35)	0.624	0.51 (0.26–0.98)	0.04
University	0.86 (0.49–1.52)	0.923	0.67 (0.25–1.79)	0.42
**Marital status**				
Single	Ref	Ref	Ref	Ref
Married	0.65 (0.31–1.36)	0.305	0.83 (0.36–3.99)	0.724
Divorced	0.18 (0.03–1.16)	0.05	0.0 (0.0–0.0)	0.999
Widowed	0.535 (0.23–1.94)	0.142	1.3 (0.39–4.3)	0.675
**Locality**				
Rural	Ref	Ref	Ref	Ref
Urban	1.2 (0.97–1.70)	0.65	1.730 (1.22–2.47)	0.002
Smoking (yes)	1.16 (0.691–1.94)	0.575	0.51 (0.20–1.2)	0.139
**BMI (kg/m** ^**2**^ **)**				
Normal or under weight	Ref	Ref	Ref	Ref
Overweight	2.32 (1.54–3.47)	<0.0001	2.44 (1.48–4.01)	<0.0001
Obese	4.0 (2.68–6.1)	<0.0001	4.78 (2.90–7.88)	<0.0001
Waist to Hip ration	1.93 (1.46–2.53)	<0.0001	2.78 (1.92–4.05)	<0.0001
History of diabetes in family	1.44 (1.07–1.93)	0.015	1.96 (1.24–3.09)	0.004
History of CVD in family	1.04 (0.71–1.52)	0.838	3.95 (2.1–7.41)	<0.0001

Note. BMI: Body mass index; CVD: cardiovascular disease.

## Discussion

This cross-sectional study was conducted in a bi-ethnic population in Naqadeh, Iran, to estimate MetS prevalence by covering more than 3000 participants. Our main findings showed that the total prevalence of MetS is 37.7%, with a higher prevalence among the Turk population (41.6%) versus the Kurd population (33.9%) and among the urban population (41.4%) versus the rural population (32.6%). Our study showed similar total MetS prevalence rates compared with other reports from Iran (22.8–42.3%)^21–23^ and other Asian countries and neighbors of Iran (16–50%)^24–27^. The wide prevalence range can be explained by the variety of populations and the times of the study in recent decades. Moreover, in our study, the Turk population had a relatively higher MetS prevalence compared with the Kurd population and other reported estimations form Iran^28^. Comparing MetS components of the Turk and Kurd populations revealed that both men and women of the Turk population had higher prevalence rates of a raised BP, abdominal obesity, a raised FBS, and TG but not low HDL. The cultural and lifestyle patterns of the Turk population may further contribute to their higher prevalence of MetS. No evidence exists regarding the level of physical activity and diet habit differences of both Turk and Kurd individuals.

An unhealthy lifestyle and dietary pattern can explain the high prevalence of MetS in urban areas^23,29^.

The prevalence of MetS and its components among women were significantly higher than those of men in both the Turk and the Kurd ethnicity except for abdominal obesity (P < 0.0001). Abdominal obesity was defined for the Iranian population as a WC ≥ 90 cm in men and women. Although the prevalence of abdominal obesity in this study was estimated based on Iranian ethnic cut-off points, making it greater than the ATP (III) definition (>102 cm for men and >88 cm for women), about half of the women in our study (49.8) had MetS. Thus, when one considers the ATP (III) criteria for abdominal obesity for women, the gender differences of MetS prevalence seem to be controversial. This result is consistent with other studies form Iran^16,24^ and other countries^30,31^ and it is inconsistent with Novak *et al*.’s results^32^, which found a higher MetS rate among men than women.

Strong associations of MetS prevalence were found with being older, being female, being overweight or obese, having a higher waist-to-hip ratio, and having a history of diabetes and CVD in the family in both the Turk and the Kurd ethnicity in the multivariate model. In our study, overweight and obese individuals had a higher risk of MetS by four and two times, respectively, among both Turks and Kurds. The prevalence of MetS increased with age and reached the highest risk at ages 70 and over among the Turk participants, with people 70 and older facing a five-times-greater risk of MetS.

### Strength and limitations

Our study is the first large community-based survey comparing MetS prevalence between two large ethnic groups of the Iranian population in the region. In addition, the findings of the study can be generalized to the population due to the large sample size and sampling procedures. However, as this study is part of the IraPEN project in Naqadeh, we have no information about the dietary habits and lifestyle of the populations.

### Conclusion and recommendations

This community-based study of the IraPEN survey identified the prevalence of MetS and its components among the two large ethnicities of Turks and Kurds in the Iranian population. The current study revealed that the prevalence of MetS and its components among the Turk people were significantly higher than those of the Kurds. Being female, living in a rural area, increasing age, being overweight or obesity, having a higher waist-to-hip ratio, and having a history of diabetes and CVD in the family were the strongest sociodemographic predictors of MetS. Due to the multiple ethnicities of the Iranian population, the recognition of the prevalence of MetS components is a major step in establishing intervention strategies for modifying cardio-metabolic risk factors based on the population’s ethnicities and their socio-demographic, cultural, and lifestyle factors. We recommend future research for planning an efficient and sustainable health education and promotion program to halt the prevalence of MetS.

## Declaration

The study was approved by the Ethics Committee of Tabriz University of Medical Sciences (IRTBZMED.REC.1396.965). All participants gave written informed consent. The data and material is available.
